# Effect of holmium laser prostatectomy on surgical outcomes of primary bladder neck obstruction

**DOI:** 10.1186/s12894-025-01693-y

**Published:** 2025-02-19

**Authors:** Hyomyoung Lee, Hyun Ju Jeong, Min Soo Choo, Sung Yong Cho, Seong Jin Jeong, Seung-June Oh

**Affiliations:** 1 Department of Urology, Seoul National University Hospital, 101 Daehak-no, Jongno-gu, Seoul, 03080 Republic of Korea; 2 Department of Urology, Seoul National University College of Medicine, Seoul, Republic of Korea; 3 Seoul Metropolitan Government-Seoul National University Boramae Medical Center, Seoul, Republic of Korea; 4 Department of Urology, Seoul National University Bundang Hospital, Seongnam-si, Gyeonggi-do Republic of Korea

**Keywords:** Bladder neck obstruction, Holmium, Laser, Prostatectomy

## Abstract

**Background:**

The purpose of this study is to evaluate the efficacy and safety of holmium laser prostatectomy in patients diagnosed with primary bladder neck obstruction (PBNO), compared with patients with benign prostatic hyperplasia (BPH).

**Methods:**

We analyzed the databases of patients who underwent a holmium laser prostatectomy or enucleation of the prostate for PBNO and BPH between January 2018 and August 2022. PBNO was diagnosed primarily based on cystourethroscopic findings. Patients were followed up according to a regular protocol at 2 weeks, 3 mo, and 6 mo postoperatively.

**Results:**

In total, 28 and 447 patients with PBNO and BPH, respectively, were identified. Preoperative urodynamic studies showed that detrusor underactivity was significantly more prevalent in the PBNO group (78.6%) than in the BPH group (57.5%) (*p* < 0.01). Both PBNO and BPH groups showed significant improvements in the total International Prostate Symptom Score, Overactive Bladder Symptom Score, and maximum flow rate at 6 mo postoperatively compared with the preoperative values (*p* < 0.01). Subjective satisfaction 6 mo after operation was not significantly different between the PBNO and BPH groups (*p* > 0.05). Complications in the PBNO group included recatheterization (*n* = 1, 3.5%), with no patients requiring blood transfusion or transurethral coagulation.

**Conclusions:**

Holmium laser prostatectomy was effective and safe for patients with PBNO, with elevated subjective patient satisfaction.

**Supplementary Information:**

The online version contains supplementary material available at 10.1186/s12894-025-01693-y.

## Introduction

Benign prostatic hyperplasia (BPH) is the most common cause of lower urinary tract symptoms (LUTS) in older men [1]. However, other causes of LUTS exist, including overactive bladder, urethral stricture, prostatitis, urinary tract infection, and neurogenic bladder dysfunction [2]. Primary bladder neck obstruction (PBNO) causes LUTS without BPH [3]. This condition is considerably rare and not fully understood by urologists [4]. To date, literature on the natural course, etiology, and presentation of PBNO is limited [5]. PBNO has not been properly established as a disease entity, leading to a significant number of misdiagnoses in clinical practice [6]. The clinical presentation of PBNO includes various symptoms such as voiding, storage, and pelvic pain and discomfort [7]. Videourodynamic study (VUDS) was considered the gold standard diagnosis for PBNO [8]. However, challenges, such as radiation exposure and high costs associated with performing VUDS to diagnose PBNO in clinical practice, exist [9]. Alpha-blockers are the first-line treatment for PBNO [10]. Surgical treatment mainly involves transurethral incision of the bladder neck [11, 12]. However, to our knowledge, no studies are available on surgical treatment for PBNO using a holmium laser. Since 2018, we have diagnosed PBNO using cystourethroscopy and treated it with a holmium laser prostatectomy. This study aimed to evaluate the efficacy and safety of holmium laser prostatectomy in patients diagnosed with PBNO compared to those diagnosed with BPH.

## Materials and methods

### Patients

This study included patients who underwent holmium laser prostatectomy or holmium laser enucleation of the prostate for PBNO and BPH at the Seoul National University Hospital between January 2018 and August 2022. Patients in both groups were managed following the same clinical protocol. This study was approved by the Institutional Review Board (IRB) of Seoul National University Hospital. (IRB No.0810-027-260, IRB NO.2407-103-1553).

**Fig. 1:**
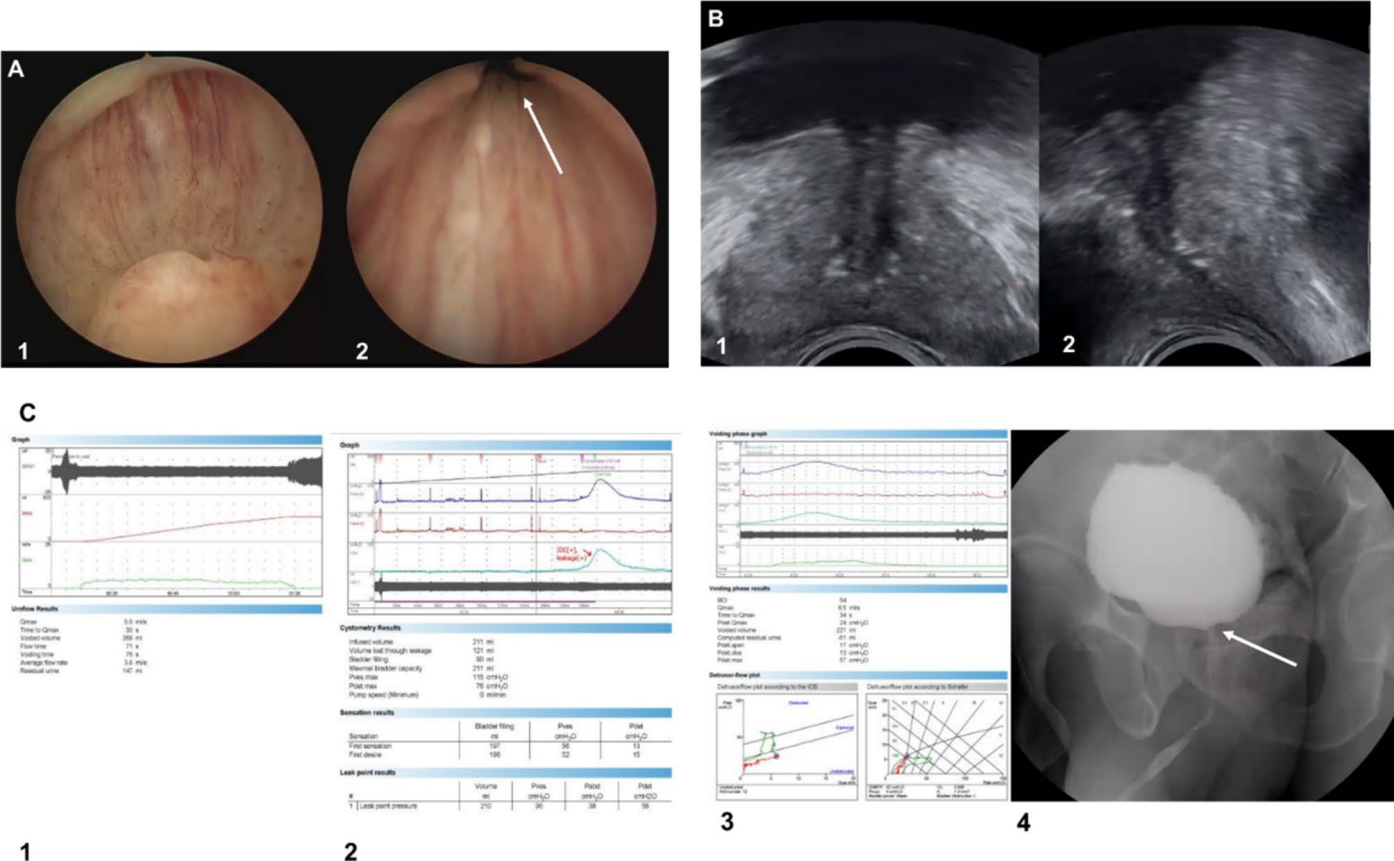
Clinical findings in a 59-year-old patient with primary bladder neck obstruction; **(A)** Cystourethroscopy in the lithotomy position with a horizontal view from the verumontanum shows that the bladder neck opening was not visible [1]. Bladder neck opening shows annular narrowing (**2**: white arrow) **(B)** Transrectal ultrasound imaging (**1**: Transverse view), (**2**: Sagittal view): Severe bladder neck elevation is noted in sagittal view. **(C)** Videourodynamic study: [1] Synnergic electromyogram (EMG) pattern of flow-EMG study and severe plateau-shaped constrictive pattern of uroflowmetry; [2] involuntary detrusor contraction on filling cystometry; [3] partly obstructive pattern of pressure-flow study; [4] bladder neck narrowing finding (white arrow) on fluoroscopic image during attempted void

**Fig. 2:**
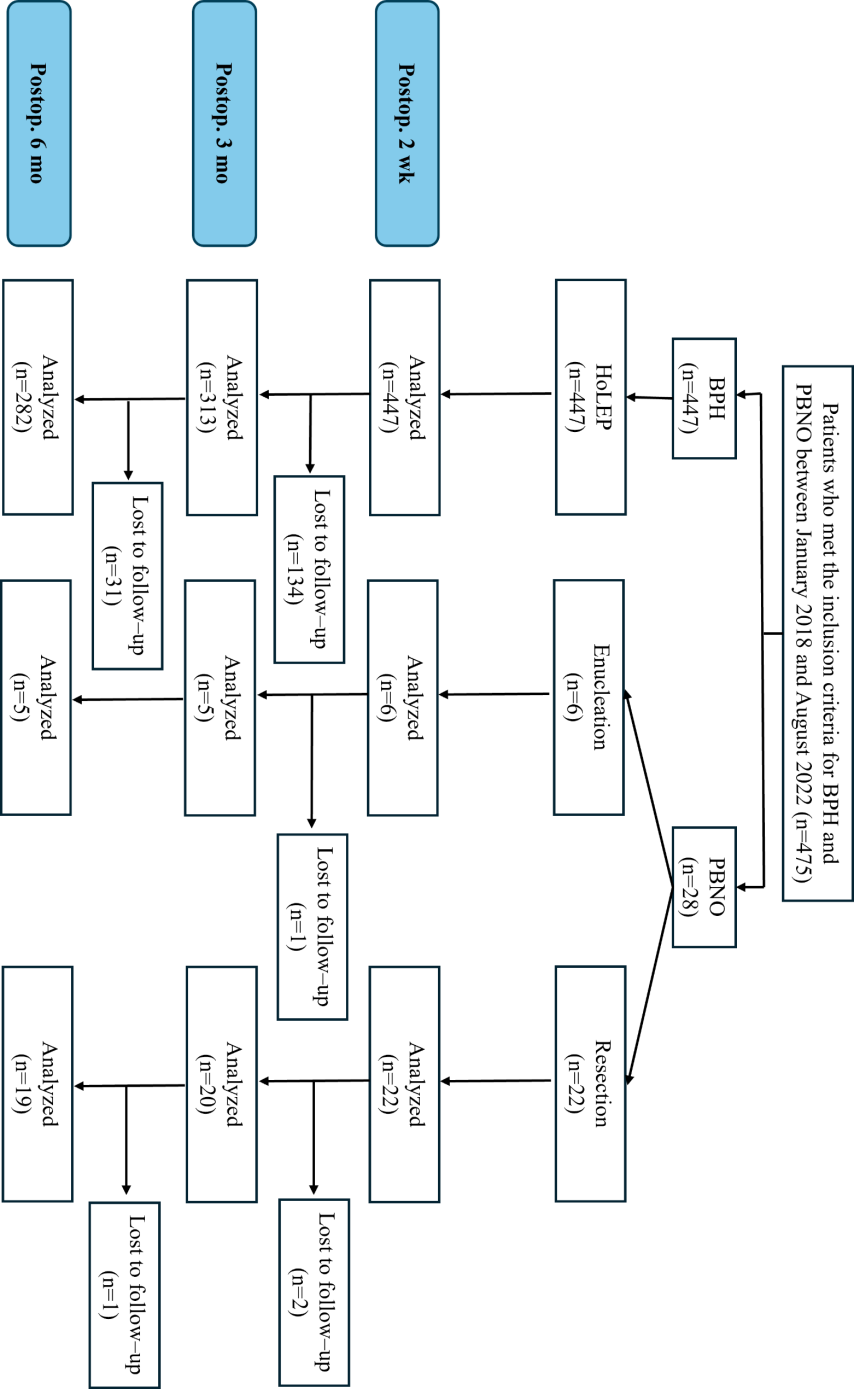
Flowchart of patient disposition

The inclusion criteria for the PBNO were as follows: Patients aged ≥ 50 y who presented with moderate to severe LUTS who visited the urology outpatient clinic; patients with typical cystourethroscopic findings for PBNO; and patients with a total prostate volume < 40 mL assessed by transrectal ultrasound (TRUS) imaging. The sagittal view on TRUS showed bladder neck elevation in most patients with PBNO (Fig. 1B). We defined typical cystourethroscopic findings for PBNO as follows: High bladder neck when viewed horizontally from the verumontanum (Fig. < link rid="fig1”>1</link>A-1); the finding of annular narrowing of the bladder neck opening (Figs. 1A and 2); isolated median lobe hypertrophy (median bar) was excluded. These findings were obtained using 30° rigid cystourethroscopy (Karl Storz Hopkins) in a lithotomy position. The inclusion criterion for the BPH group was patients aged ≥ 50 y, with clinical diagnosis of BPH. The exclusion criteria for the PBNO and BPH groups were the presence of genitourinary cancer, history of surgery, urethral stricture, urinary tract infection (UTI), interstitial cystitis, and neurogenic bladder dysfunction. Patients with minimal neuropathy, which was determined to have a negligible or minimal impact on LUTS by medical history and physical examination, were included in this study.

## Study design

The diagnostic workup included a physical examination, including digital rectal examination, assessment of symptoms using the International Prostate Symptom Score (IPSS) and Overactive Bladder Symptom Score (OABSS), urinalysis, and urine culture to exclude UTI, TRUS to measure prostate volume, uroflowmetry with ultrasound measurement of post-void residual urine volume and prostate-specific antigen (PSA) test. In cases where nodules were palpable on DRE or elevated PSA levels clinically indicated suspicion of prostate cancer, a TRUS-guided prostate biopsy was performed. After confirming a negative result for prostate cancer in the pathological report, surgery was performed on different dates. A urodynamic study (UDS) was performed on all patients [13]. The bladder contractility index (BCI) was defined as PdetQmax + 5Qmax [14]. We defined patients with a BCI < 100 as having detrusor underactivity (DUA) and those with a BCI of ≥ 100 as having non-DUA [15].

The surgical outcomes included operative time, enucleation time, morcellation time, and extracted prostate volume. Perioperative outcomes included the duration of Foley catheterization, length of hospital stay after surgery, and surgical pathology. The IPSS, OABSS, and uroflowmetry data were measured at 2 weeks, 3 mo, and 6 mo postoperatively. Patient-reported subjective satisfaction with the surgical outcomes was assessed 6 mo postoperatively [16]. Postoperative complications were evaluated at 2 weeks, 3 mo, and 6 mo using the Clavien-Dindo classification.

## Surgical methods

The patient was placed in the lithotomy position under spinal or general anesthesia. The Ho: YAG laser (VersaPulse PowerSuite 100 W, Lumenis Pulse™ 120 H, Yokneam, Israel) was set to 80 W (2 J, 40 Hz). The three-lobe technique is primarily used when a clear surgical plane for enucleation is identified [17, 18]. The median lobe was first enucleated. Initial incisions were made on both sides of the verumontanum to identify the capsular plane. The surgical plane of capsule is characterized by circular fibers running in the transverse direction. Longitudinal incisions were made at the 5 o’clock and 7 o’clock positions of the bladder neck, connecting with the previous incisions. Transverse incision was made immediately above the verumontanum to enucleate the median lobe.

Resection of both lateral lobe was performed for cases in which the surgical plane in the lateral lobes was not identifiable during enucleation. Resection of each lateral lobe was then started distally at the verumontanum, and the lower limit was defined with incisions on both sides of the initial incision at the verumontanum. A prostatic mucosal incision was performed at the 1 and 11 o’clock positions over the entire length of the prostate to define the margin of the lateral lobe resection. The lobe was then released, starting distally, until only a 12 o’clock mucosal bridge remained at the bladder neck [19]. After meticulous bleeding control in the prostatic fossa, morcellation was performed using a 26-Fr nephroscope and a tissue morcellator (Versacut™, Lumenis). A 22-Fr 3-way Foley catheter was placed under continuous irrigation and removed on the first postoperative day. The patients were discharged typically on the first day after surgery.

### Statistical analysis

All variables were expressed as mean ± standard deviation. Propensity matching was performed when a significant difference was observed in the sample size between the BPH and PBNO groups. For the comparison of clinical parameters between the two groups, paired t-tests were used for continuous variables and chi-square tests for categorical variables. Within each group, changes in postoperative functional outcomes were compared using paired t-tests and chi-squared tests for continuous and categorical variables, respectively. Statistical significance was set at *p* < 0.05.

## Results

### Patient demographics and operative and perioperative outcomes

Twenty-eight patients with PBNO and 447 with BPH were identified (Table 1) (Fig. 2). The mean age of the PBNO group was 67.9 (± 6.5) y, and the mean total prostate volume was 32.0 (± 8.8) mL. No significant differences were observed in the baseline total IPSS and OABSS between the PBNO and BPH groups (*p* = 0.47, *p* = 0.38). On preoperative UDS, detrusor underactivity was significantly more prevalent in the PBNO group (78.6%) than in the BPH group (57.5%) (*p* < 0.01). The total operation time was shorter in the PBNO group [26.7 (± 9.5) min] than in the BPH group [61.4 (± 32.0) min] (*p* < 0.01). The Bladder Outlet Obstruction Index in the BPH group and the PBNO group was 38.4 (± 15.9) and 30.7 (± 15.9), respectively, showing no significant difference (*p* = 0.16).

**Table 1:** Baseline patient demographics, operative, and perioperative outcomes

Characteristics	BPH	PBNO	*P*-value*
	(*n* = 447)	(*n* = 28)	
**Baseline characteristics**			
Age (years, mean ± SD)	68.9 (± 6.6)	67.9 (± 6.5)	0.79
Comorbidities			
Diabetes Miletus (n,%)	89 (19.9%)	5 (18.0%)	0.44
Hypertension (n,%)	127 (28.4%)	7 (25.0%)	0.37
Cardiovascular disease (n,%)	20 (4.4%)	2 (7.0%)	0.21
Preoperative PSA level (ng/mL, mean ± SD)	3.9 (± 4.1)	0.9 (± 0.8)	0.08
Total prostate volume (mL, mean ± SD)	65.6 (± 27.4)	32.0 (± 8.8)	**< 0.01**
Prostate transition zone volume (mL, mean ± SD)	37.1 (± 21.7)	10.3 (± 4.2)	**< 0.01**
IPSS (International Prostate Symptom Score)			
IPSS, storage symptom score ( ± SD)	7.0 (± 2.9)	8.2 (± 4.2)	0.32
IPSS, voiding symptom score (mean ± SD)	12.7 (± 4.5)	13.4 (± 5.0)	0.77
IPSS, total score (mean ± SD)	19.7 (± 6.8)	21.6 (± 8.4)	0.47
IPSS, QoL score (mean ± SD)	4.0 (± 1.2)	4.2 (± 1.0)	0.56
OABSS questionnaire score (mean ± SD)	5.6 (± 2.6)	6.7 (± 3.9)	0.38
Qmax (mL/sec, mean ± SD)	11.3 (± 4.9)	8.9 (± 2.8)	0.22
Post-void residual volume (mL, mean ± SD)	98.7 (± 90.8)	47.2 (± 52.1)	0.06
Pre operative urodynamics			
Bladder capacity (mL, mean ± SD)	278.6 (± 156.8)	253.8 (± 152.9)	0.64
Bladder complicance (mL/cmH2O, mean ± SD)	60.6 (± 35.5)	62.8 (± 22.3)	0.81
Qmax (mL/sec, mean ± SD)	8.3 (± 3.6)	5.9 (± 1.4)	**< 0.01**
PdetQmax (cmH2O, mean ± SD)	54.9 (± 12.5)	42.6 (± 15.9)	**< 0.01**
Bladder contractility index^*^	96.2 (± 19.0)	72.3 (± 17.9)	**< 0.01**
Bladder outlet obstruction index^*^	38.4 (± 15.9)	30.7 (± 15.9)	0.16
Detrusor underactivity (n,%)	257 (57.5%)	22 (78.6%)	**< 0.01**
**Operative outcomes**			
Total operation time (min, mean ± SD)	61.4 (± 32.0)	26.7 (± 9.5)	**< 0.01**
Enucleation or resection time (min, mean ± SD)	36.6 (± 16.6)	13.3 (± 5.1)	**< 0.01**
Morcellation time (min, mean ± SD)	12.8 (± 9.2)	2.4 (± 2.2)	**< 0.01**
Extracted tissue volume (ml, mean ± SD)	25.5 (± 18.7)	3.0 (± 3.4)	**< 0.01**
**Perioperative Outcomes**			
Catheterization time (day, mean ± SD)	1.9 (± 2.3)	1.4 (± 3.7)	0.37
Postoperative hospital stay (day, mean ± SD)	1.2 (± 1.1)	1.0 (± 0.0)	0.44
Surgical pathology			
Benign nodular hyperplasia (n,%)	423 (94.6%)	27 (96.0%)	0.52
Incidental prostate adenocarcinoma (n,%)	24 (5.4%)	1 (4.0%)	0.65

SD, standard deviation; BPH, Benign prostatic hyperplasia; PBNO, Primary bladder neck obstruction; PSA, prostate-specific antigen; IPSS, International Prostate Symptom Score; QoL, quality of life; OABSS, Overactive Bladder Symptom Score; Qmax, maximum flow rate; PdetQmax, Maximum detrusor pressure at Qmax; *P-value represents comparison between the PBNO and BPH groups

## Efficacy

**Fig. 3:**
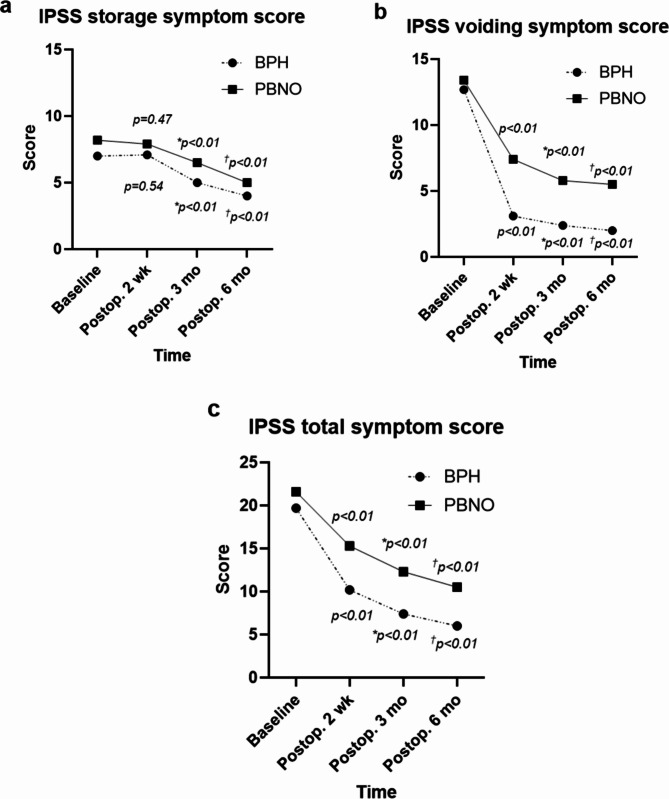
International prostate symptom score (IPSS) according to the postoperative follow-up period. **(a)** IPSS storage score; **(b)** IPSS voiding score; **(c)** IPSS total score. p-value indicates the difference in IPSS score between baseline and postoperative 2 weeks. *p-value indicates the difference in IPSS score between baseline and postoperative 3 months. †p-value indicates the difference in IPSS score between baseline and postoperative 6 months

The postoperative functional outcomes and results of the three self-administered questionnaires for the PBNO and BPH groups are presented in Table 2 and Fig. 3. The total IPSS significantly improved at 2 weeks postoperatively compared to the preoperative values in both the PBNO and BPH groups (*p* < 0.01), whereas no significant differences were observed in the OABSS between the preoperative and 2-week postoperative assessments (*p* = 0.27, *p* = 0.32).

**Table 2:** Postoperative functional outcomes and results of the three self-administered questionnaires

Variable	BPH (*n* = 447)	*P*-value*	PBNO (*n* = 28)	*P*-value*	*P*-value**
**Baseline**					
IPSS, storage symptom score (mean ± SD)	7.0 (± 2.9)	-	8.2 (± 4.2)	-	0.32
IPSS, voiding symptom score (mean ± SD)	12.7 (± 4.5)	-	13.4 (± 5.0)	-	0.77
IPSS, total score (mean ± SD)	19.7 (± 6.8)	-	21.6 (± 8.4)	-	0.47
IPSS, QoL score (mean ± SD)	4.0 (± 1.2)	-	4.2 (± 1.0)	-	0.56
OABSS^#^ (mean ± SD)	5.6 (± 2.6)	-	6.7 (± 3.9)	-	0.38
Q_max_ (mL/sec) (mean ± SD)	11.3 (± 4.9)	-	8.9 (± 2.8)	-	0.22
Post-void residual volume (mL) (mean ± SD)	98.7 (± 90.8)	-	47.2 (± 52.1)	-	0.06
**Postoperative 2weeks**					
IPSS, storage symptom score (mean ± SD)	7.1 (± 3.2)	0.54	7.9 (± 3.5)	0.47	0.45
IPSS, voiding symptom score (mean ± SD)	3.1 (± 2.4)	**< 0.01**	7.4 (± 6.8)	**< 0.01**	0.06
IPSS, total score (mean ± SD)	10.2 (± 4.7)	**< 0.01**	15.3 (± 9.3)	**< 0.01**	0.08
IPSS, QoL score (mean ± SD)	2.6 (± 1.1)	**< 0.01**	3.0 (± 1.6)	**< 0.01**	0.07
OABSS^#^ (mean ± SD)	6.2 (± 2.8)	0.27	6.3 (± 3.9)	0.32	0.89
**Postoperative 3mo**					
IPSS, storage symptom score (mean ± SD)	5.0 (± 2.9)	**< 0.01**	6.5 (± 3.8)	**< 0.01**	0.19
IPSS, voiding symptom score (mean ± SD)	2.4 (± 2.9)	**< 0.01**	5.8 (± 4.6)	**< 0.01**	**0.02**
IPSS, total score (mean ± SD)	7.4 (± 4.7)	**< 0.01**	12.3 (± 7.9)	**< 0.01**	0.06
IPSS, QoL score (mean ± SD)	2.1 (± 1.3)	**< 0.01**	2.8 (± 1.7)	**< 0.01**	0.06
OABSS^#^ (mean ± SD)	5.2 (± 2.7)	**< 0.01**	6.0 (± 3.6)	**< 0.01**	0.44
Q_max_ (mL/sec) (mean ± SD)	26.2 (± 12.3)	**< 0.01**	16.8 (± 8.0)	**< 0.01**	**0.02**
Post-void residual volume (mL) (mean ± SD)	21.9 (± 27.3)	**< 0.01**	12.0 (± 18.6)	**< 0.01**	0.50
**Postoperative 6mo**					
IPSS, storage symptom score (mean ± SD)	4.0 (± 2.8)	**< 0.01**	5.0 (± 3.6)	**< 0.01**	0.36
IPSS, voiding symptom score (mean ± SD)	2.0 (± 3.4)	**< 0.01**	5.5 (± 4.8)	**< 0.01**	**0.01**
IPSS, total score (mean ± SD)	6.0 (± 4.7)	**< 0.01**	10.5 (± 7.9)	**< 0.01**	0.07
IPSS, QoL score (mean ± SD)	1.7 (± 1.3)	**< 0.01**	2.1 (± 1.6)	**< 0.01**	0.10
OABSS^#^ (mean ± SD)	3.3 (± 2.5)	**< 0.01**	3.4 (± 2.3)	**< 0.01**	0.81
Q_max_ (mL/sec) (mean ± SD)	23.6 (± 5.6)	**< 0.01**	14.8 (± 5.6)	**< 0.01**	**0.01**
Post-void residual volume (mL) (mean ± SD)	28.3 (± 35.9)	**< 0.01**	18.5 (± 15.9)	**< 0.01**	0.98
**Self-administered questionnaires**					
Satisfaction with treatment question (STQ)					0.087
- Satisfied	412 (92.2%)	-	17 (60.7%)	-	
- Neutral	13 (2.9%)	-	4 (14.3%)	-	
- Unsatisfied	22 (4.9%)	-	7 (25.0%)	-	
Overall response assessment (ORA)					0.566
- Improved	420 (94.0%)	-	23 (82.1%)	-	
- Neutral	25 (5.6%)	-	5 (17.9%)	-	
- Aggravated	2 (0.4%)	-	0 (0.0%)	-	
Willingness to undergo surgery question (WSQ)					0.093
- Definitely	395 (88.4%)	-	15 (53.6%)	-	
- Neutral	34 (7.6%)	-	10 (35.7%)	-	
- Not likely	18 (4.0%)	-	3 (10.7%)	-	

SD, standard deviation; BPH, Benign prostatic hyperplasia; PBNO, Primary bladder neck obstruction; IPSS, International Prostate Symptom Score; QoL, quality of life; OABSS, Overactive Bladder Symptom Score; Qmax, maximum flow rate; * P-value represents comparison between baseline results; ** P-value represents comparison between the PBNO and BPH groups

The PBNO and BPH groups showed significant improvements in total IPSS, OABSS, and Qmax at 3 and 6 mo postoperatively compared with preoperative values (*p* < 0.01). However, the PBNO group exhibited less improvement in the IPSS voiding score and maximum flow rate (Qmax) at 3 and 6 mo postoperatively than the BPH group (*p* < 0.01). Additionally, at 6 mo postoperatively, the total IPSS was higher in the PBNO group [10.5 (± 7.9)] than in the BPH group [6.0 (± 4.7)] (*p* = 0.07). No significant differences were observed in OABSS at 6 mo postoperatively between the PBNO group [3.4 (± 2.3)] and the BPH group [3.3 (± 2.5)] (*p* = 0.81).

At 6 mo postoperatively, the proportion of patients in the BPH group who responded positively to the satisfaction with treatment question (STQ) was higher than that in the PBNO group; however, this difference was not statistically significant (STQ: 60.7% vs. 92.2%, *p* = 0.087). Similarly, a higher proportion of patients in the BPH group showed a positive response to the overall response assessment (ORA) and the willingness to undergo surgery question (WSQ) compared to the PBNO group; however, there were no statistical differences [ORA: 82.1% vs. 94.0%, *p* = 0.566; WSQ: 53.6% vs. 88.4%, *p* = 0.093].

## Safety

The PBNO group showed one case of recatheterization at 2 weeks postoperatively (*n* = 1, 3.5%) (Table 3). None of the patients required a blood transfusion or transurethral coagulation. During the follow-up period of up to 6 months postoperatively, there were no complications of bladder neck contracture or urethral stricture in both groups.

**Table 3:** Postoperative complications

Characteristics	Clavien-Dindo grade	BPH (*n* = 447)	PBNO (*n* = 28)	*P*-value*
**Postoperative 2 weeks**				
Blood transfusion (n,%)	II	2 (0.4%)	0 (0.0%)	0.31
Transurethral coagulation (n,%)	IIIB	0 (0.0%)	0 (0.0%)	-
Re-catheterization (n,%)	II	17 (3.8%)	1 (3.5%)	0.61
Stress urinary incontinence (n,%)	II	28 (6.2%)	1 (3.5%)	0.23
Urgency urinary incontinence (n,%)	II	23 (5.1%)	2 (7.1%)	0.46
**Postoperative 3 mo**				
Stress urinary incontinence (n,%)	II	20 (4.5%)	0 (0.0%)	0.47
Urgency urinary incontinence (n,%)	II	18 (4.0%)	1 (3.5%)	0.51
**Postoperative 6 mo**				
Stress urinary incontinence (n,%)	II	0 (0.0%)	0 (0.0%)	-
Urgency urinary incontinence (n,%)	II	0 (0.0%)	0 (0.0%)	-
Bladder neck contracture (requiring TUI) (n,%)	IIIB	0 (0.0%)	0 (0.0%)	-
Urethral stricture (de novo) (n,%)	IIIA	0 (0.0%)	0 (0.0%)	-
Prostatic fossa stone (requiring cystoscopic stone removal) (n,%)	IIIB	0 (0.0%)	0 (0.0%)	-

BPH, Benign prostatic hyperplasia; PBNO, Primary bladder neck obstruction; * P-value represents comparison between the PBNO and BPH

## Discussion

PBNO is characterized by the bladder neck not opening sufficiently during urination, leading to obstructed urinary flow without any anatomical obstruction, such as benign prostate enlargement or urethral stenosis [6, 7].

There is no universal agreement or diagnosis for PBNO. Traditionally, diagnosis is achieved by coupling the outcomes of UDS with radiographic visualization of the bladder neck area (Fig. 1C) [5, 8]. The urodynamic outcome is characterized by outlet obstruction accompanied by a reduction in Qmax (10–15 mL/s; normal value > 18 mL/s), high-pressure detrusor contractility, and increased intravesical pressure [20]. Nitti et al. categorized PBNO into the following three distinct types: (1) classic high-pressure, low-flow voiding; (2) normal-pressure, low-flow voiding with narrowing of the bladder neck; and (3) delayed opening of the bladder neck. These three classifications indicate vesical neck dysfunction, resulting in functional obstruction [21]. Other previous studies [4, 22] have characterized the urodynamic outcomes of PBNO. However, no universally accepted definition of the video-UDS for PBNO exists. Furthermore, video-UDS is limited by its low availability, high cost, and radiation exposure [9].

Girolamo et al. attempted to diagnose PBNO using MR voiding cystourethrography to overcome the limitations of the video-UDS [20]. MR voiding cystourethrography offers advantages such as reduced radiation exposure and elimination of the need for cannulation maneuvers of the urethra [20]. However, a limitation of MR voiding cystourethrography is that the diagnostic tool is unfamiliar to urologists and requires a specialist. In contrast, cystourethroscopy has advantages over video-UDS regarding accessibility and cost, and it is more familiar to urologists. In this study, PBNO was diagnosed when the bladder neck was not visible when viewed horizontally from the verumontanum using rigid cystourethroscopy (Fig. < link rid="fig1”>1</link>A-1). The bladder neck opening shows annular narrowing (Figs. 1A and 2). Using the criteria for diagnosing PBNO with cystourethroscopy, as outlined in this study, could help urologists to diagnose PBNO in their clinical practice.

To our knowledge, only three studies have reported transurethral incision (TUI) for PBNO. Kochakarn et al. performed a unilateral TUI of the bladder neck in patients with PBNO (*n* = 35) [11]. Follow-up was conducted for up to 1 y postoperatively, and their retrospective analysis showed that the IPSS and Qmax significantly improved. Yang et al. conducted a prospective study (*n* = 33) after TUI of the bladder neck, with preservation of the supramontanal tissue [23]. IPSS and Qmax significantly improved 2 y postoperatively. Mattioli et al. performed a retrospective analysis (*n* = 196) of TUI using a thulium laser. The IPSS and Qmax significantly improved 1 y postoperatively [12]. The authors’ study was followed up for 6 mo postoperatively. The total IPSS score and Qmax improved compared to those before surgery and were similar to those in the postoperative outcomes of previous studies [11, 12, 23].

We performed a holmium laser prostatectomy on patients with PBNO; to our knowledge, this is the first study using this surgical method. Holmium laser prostatectomy was performed instead of TUI because of the possibility of recurrence. According to the authors’ previous surgical experience of performing a TUI of the bladder neck in patients diagnosed with PBNO, the functional outcome improved postoperatively; however, symptoms of obstruction recurred during long-term follow-up. On cystourethroscopy, the bladder neck remained in the form of an isolated median lobe because of the previous incision. Accordingly, a secondary prostatectomy was performed to remove the enlarged median lobe. No recurrence of symptoms occurred thereafter. Based on our experience with these cases, we have been performing a holmium laser prostatectomy rather than TUI of the bladder neck in patients with PBNO since 2018.

No previous studies have compared the results of surgical treatment for PBNO with those for BPH. In this study, the PBNO group showed less improvement in the IPSS voiding score [5.5 (± 4.8) vs. 2.0 (± 3.4)] and Qmax [14.8 (± 5.6) mL/s vs. 23.6 (± 5.6) mL/s] at 6 mo after surgery compared to the BPH group. In the subjective satisfaction survey 6 mo postoperatively, the PBNO group showed lower satisfaction than the BPH group, although the difference was not statistically significant. The difference in the results of the objective and subjective indicators after surgery between the PBNO and BPH groups was attributed to the fact that the ratio of the DUA among the UDS parameters of the PBNO and BPH groups performed before surgery was higher in the PBNO group (78.6%) than in the BPH group (57.5%). The underlying mechanisms responsible for the observed increased prevalence of preoperative DUA in the PBNO group compared to the BPH group remain unclear, necessitating further investigation.

The advantages of this study are as follows: First, unlike previous studies, we compared and analyzed the postoperative outcomes in the PBNO and BPH groups. Second, the two patient groups were registry-based prospective cohorts that included patients who underwent diagnosis and treatment according to the same clinical protocol. This study had some limitations. First, mid-term follow-up was performed until 6 mo postoperatively, and long-term follow-up was not possible. Second, the number of cases in the PBNO group (*n* = 28) was relatively small compared with that in the BPH group. Third, preoperative and postoperative sexual function was not assessed, which requires investigation in future studies.

## Conclusions

Holmium laser prostatectomy was effective and safe for patients with PBNO with elevated subjective patient satisfaction.

## Electronic supplementary material

Below is the link to the electronic supplementary material.

Supplementary Material 1

## Data Availability

The authors fully understand that the better the journal, the more likely it is to releaseraw patient data to the public to increase data availability. The authors have sought the opinion of theSeoul National University Hospital IRB regarding the public sharing of data related to this study. Theauthors concluded that our raw data cannot be shared publicly for the following two reasons. 1)‘Opening this data to the public’ means ‘exporting the data to the outside.’ In Korea, the PersonalInformation Protection Act has been gradually strengthened recently. The most recent PersonalInformation Protection Act of Korea states that “the consent from the legally relevant individual subjectmust be obtained when providing personal information to a third party” and “Personal informationshould not be used beyond the scope of the purpose for which it was collected or provided to a thirdparty.” The release of raw data to the public was not included in the consent forms we received fromthe patients or in the IRB approval for this study. 2) The PBNO patient group is very small (28 patients), so even if the personal information of the patients is de-identified, there is a risk that they can be easilyidentified. For the above reasons, the authors regret that they cannot share the raw data with the publicand ask for your generous understanding.

## Abbreviations

BCI: Bladder contractility index
BPH: Benign prostatic hyperplasia
DUA: Detrusor underactivity
IPSS: International prostate symptom score
IRB: Institutional review board
LUTS: Lower urinary tract symptoms
OABSS: Overactive bladder symptom score
ORA: Overall response assessment
PBNO: Primary bladder neck obstruction
PSA: Prostate-specific antigen
Qmax: Maximum flow rate
STQ: Satisfaction with treatment question
TRUS: Transrectal ultrasound
TUI: Transurethral incision
UDS: Urodynamic study
UTI: Urinary tract infection
VUDS: Video urodynamic study
WSQ: Willingness to undergo surgery question
